# The Transcription Regulator Patz1 Is Essential for Neural Stem Cell Maintenance and Proliferation

**DOI:** 10.3389/fcell.2021.657149

**Published:** 2021-04-07

**Authors:** Sara Mancinelli, Michela Vitiello, Maria Donnini, Francesca Mantile, Giuseppe Palma, Antonio Luciano, Claudio Arra, Laura Cerchia, Giovanna Lucia Liguori, Monica Fedele

**Affiliations:** ^1^Institute of Genetics and Biophysics, National Research Council, Naples, Italy; ^2^Department of Biomedical Sciences, Humanitas University, Pieve Emanuele, Italy; ^3^Istituto di Ricovero e Cura a Carattere Scientifico (IRCCS) Humanitas Research Hospital, Rozzano, Italy; ^4^Institute for Experimental Endocrinology and Oncology, National Research Council, Naples, Italy; ^5^Struttura Semplice Dipartimentale (S.S.D.) Sperimentazione Animale, Istituto Nazionale Tumori—Istituto di Ricovero e Cura a Carattere Scientifico (IRCCS)–Fondazione G. Pascale, Naples, Italy

**Keywords:** neurogenesis, neural stem cells, PATZ1, knockout mice, neurosphere assay, subventricular zone

## Abstract

Proper regulation of neurogenesis, the process by which new neurons are generated from neural stem and progenitor cells (NS/PCs), is essential for embryonic brain development and adult brain function. The transcription regulator *Patz1* is ubiquitously expressed in early mouse embryos and has a key role in embryonic stem cell maintenance. At later stages, the detection of *Patz1* expression mainly in the developing brain suggests a specific involvement of *Patz1* in neurogenesis. To address this point, we first got insights in *Patz1* expression profile in different brain territories at both embryonic and postnatal stages, evidencing a general decreasing trend with respect to time. Then, we performed *in vivo* and *ex vivo* analysis of *Patz1*-knockout mice, focusing on the ventricular and subventricular zone, where we confirmed *Patz1* enrichment through the analysis of public RNA-seq datasets. Both embryos and adults showed a significant reduction in the number of *Patz1*-null NS/PCs, as well as of their self-renewal capability, compared to controls. Consistently, molecular analysis revealed the downregulation of stemness markers in NS/PCs derived from *Patz1*-null mice. Overall, these data demonstrate the requirement of Patz1 for NS/PC maintenance and proliferation, suggesting new roles for this key transcription factor specifically in brain development and plasticity, with possible implications for neurodegenerative disorders and glial brain tumors.

## Introduction

The Poxviruses and Zinc finger (POZ)/Broad complex, Tramtrack, and Bric à brac (BTB), and AT-hook containing Zinc finger protein 1 (PATZ1), also referred as MAZ related factor (MAZR), Zinc finger Sarcoma gene (ZSG) or Zinc finger factor/protein (ZNF278/Zfp278), is a transcriptional regulator that modulates the expression of several genes, either negatively or positively depending on the cellular context (Fedele et al., 2000, 2017; Valentino et al., 2013a). PATZ1 belongs to the POZ and Krüppel (POK) family of transcription factors, which are implicated in many biological processes, including B cell fate determination, cell cycle progression, DNA damage responses and development (gastrulation, limb formation, hematopoietic stem cell fate determination) (Kelly and Daniel, 2006; Costoya, 2007). Accordingly, Patz1 has been implicated in T cell differentiation (Ellmeier, 2015; Andersen et al., 2019; Orola et al., 2019), cell cycle regulation (Valentino et al., 2013b), DNA damage response (Keskin et al., 2015), and embryonic development (Valentino et al., 2013b). Noteworthy, a critical role for Patz1 has been described in the maintenance of embryonic stemness through its functional interaction with the pluripotency master genes *Nanog* and *Pou5f1*, which are both targets and regulators of Patz1 (Ow et al., 2014). Consistently, mouse embryonic fibroblasts lacking Patz1 show low reprogramming efficiency in induced pluripotent stem cells (iPSCs) (Ma et al., 2014).

*In situ* hybridization analysis demonstrated that *Patz1* gene is actively expressed during early embryogenesis in several districts including the central nervous system, where it mainly localizes in proliferating neural progenitors (NPCs) of the periventricular neocortical neuroepithelium, suggesting a role for Patz1 in neurogenesis (Valentino et al., 2013b). Indeed, the ventricular and subventricular zone (VZ-SVZ) is located in the wall of the lateral brain ventricles and produces new neurons in both embryonic and postnatal brain of mammals. In the embryonic brain, immature, and neurogenic transient-amplifying NPCs, the intermediate progenitor cells (IPCs), originated from multipotent radial glia cells (RGCs), also known as apical progenitors (APs) in the VZ-SVZ (Gaspard et al., 2008; Okamoto et al., 2016; Jabaudon, 2017; Telley et al., 2019). The IPCs produce a huge variety of neuronal cell types that migrate toward their final destinations, thus contributing to brain formation and function (Mancinelli and Lodato, 2018). In the adult, neural stem/progenitor cells (NS/PCs) are also present in the SVZ. NSCs activate, upon an injury or inflammatory stimulus, to produce transient-amplifying neural progenitor cells (NPCs) that divide and generate a different type of proliferating progenitors, the neuroblasts (NBs), that eventually differentiate to produce newborn neurons (Alvarez-Buylla and Garcia-Verdugo, 2002; Chaker et al., 2016; Belenguer et al., 2021).

Genetic inactivation of *Patz1* gene in mice results in a partial embryonic lethality and several developmental defects in testes, cardiac outflow tract and brain (Fedele et al., 2008; Valentino et al., 2013b). The latter defects consist in hypoproliferation of the periventricular tissue, one of the key neurogenic sites, consistently with the idea that Patz1 could play a crucial role in neurogenesis (Valentino et al., 2013b).

To verify this hypothesis, we first deeply examined *Patz1* gene expression in different sub-regions of the embryonic, fetal, juvenile and adult mouse brain, showing a decreasing trend with respect to time, as opposed to a stable expression of *Patz1* at neurogenic sites of the adult brain. Then, we analyzed the *in vivo* incorporation of the DNA intercalant 5′-bromo-2′-deoxyuridine (BrdU) at embryonic stage, and we isolated and *ex vivo* cultured both embryonic and adult NS/PCs from *Patz1*-null homozygotes, heterozygotes and *wild type* mice to perform neurosphere assay and gene expression analysis. All together the results indicate that *Patz1* inactivation leads to a drastic impairment in the number and self-renewal capacity of NS/PCs, therefore pointing out to a key role of Patz1 in maintenance and proliferation of NS/PCs.

## Materials and Methods

### Analysis of *Patz1* Transcript Distribution From Publicly Available Datasets

**FIGURE 1:**
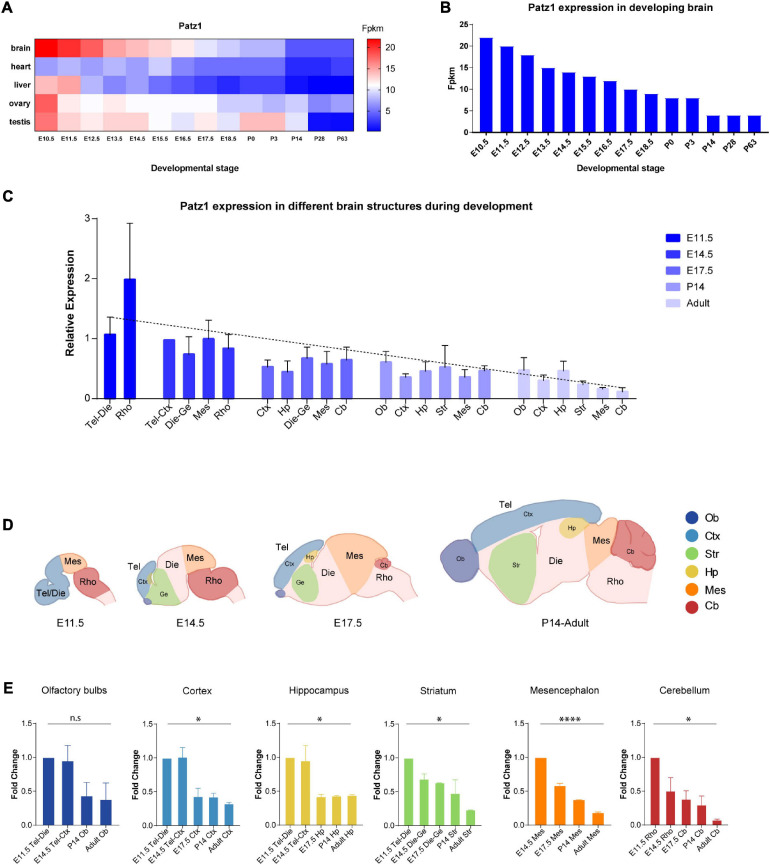
Patz1 expression in embryonic and postnatal tissues and brain structures. **(A)** Heatmap showing *Patz1* expression from EMBL-EBI Expression Atlas in the murine brain, heart, liver, testis, and ovary across development. **(B)** Bar plot highlighting *Patz1* expression levels in the brain during development from EMBL-EBI Expression Atlas. **(C)** Bar plot showing the relative expression of *Patz1* (2^–ΔΔ*Ct*^) in different embryonic, postnatal and adult brain regions, using the E14.5 Tel-Die sample as calibrator. **(D)** Schematic representation of brain structures at different developmental stages. **(E)** Bar plots showing *Patz1* fold change expression relative to E11.5 or E14.5 control sample (2^–ΔΔ*Ct*^) in the different microdissected brain structures. Tel, Telencephalon; Die, Diencephalon; Mes, Mesencephalon; Rho, Rhombencephalon; Ob, Olfactory bulbs; Ctx; Cerebral Cortex; Hp, Hippocampus; Ge, Ganglionic eminence; Cb, Cerebellum; Str, Striatum. Graphs are represented as mean values ± SD and all the significant differences are indicated by asterisks. ^∗^*p* < 0.05; ^*⁣*⁣**^p < 0.0001, as assessed by ANOVA. n.s. = not significant.

**FIGURE 2:**
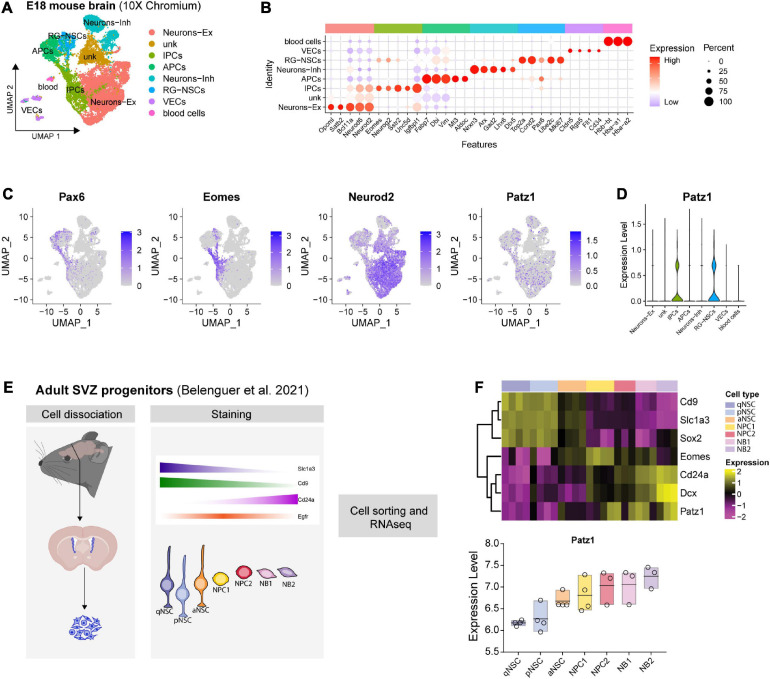
*Patz1* expression in embryonic and adult neural stem/progenitor cells. **(A)** UMAP plot showing the cell type composition of E18 mouse brain and **(B)** relative diagnostic cell marker enrichment in term of expression and percent of positive cells. **(C)** Feature plot of selected genes: *Pax6*, *Eomes (Tbr2)*, *Neurod2*, *Patz1.* **(D)** Violin plot showing *Patz1* enrichment in Radial Glia and Intermediate Progenitor cell types. **(E)** Experimental scheme used by Belenguer et al. (2021) to isolate different neural stem/progenitor cells from adult murine SVZ. **(F)** Heatmap and box plot showing the expression of *Patz1* and selected NS/PCs markers in adult murine SVZ. RG, Radial Glia; NSC, Neural Stem Cells; IPC, Intermediate Progenitor Cells; APC, Apical Progenitor Cell; VEC, vascular endothelial cell; Ex, Excitatory; Inh, Inhibitory; qNSC, quiescent Neural Stem Cell; pNSC, primed Neural Stem Cells; aNSC, activated Neural Stem Cells; NPC, Neural Progenitor Cell; NB, Neuroblast; SVZ, Subventricular Zone.

**FIGURE 3:**
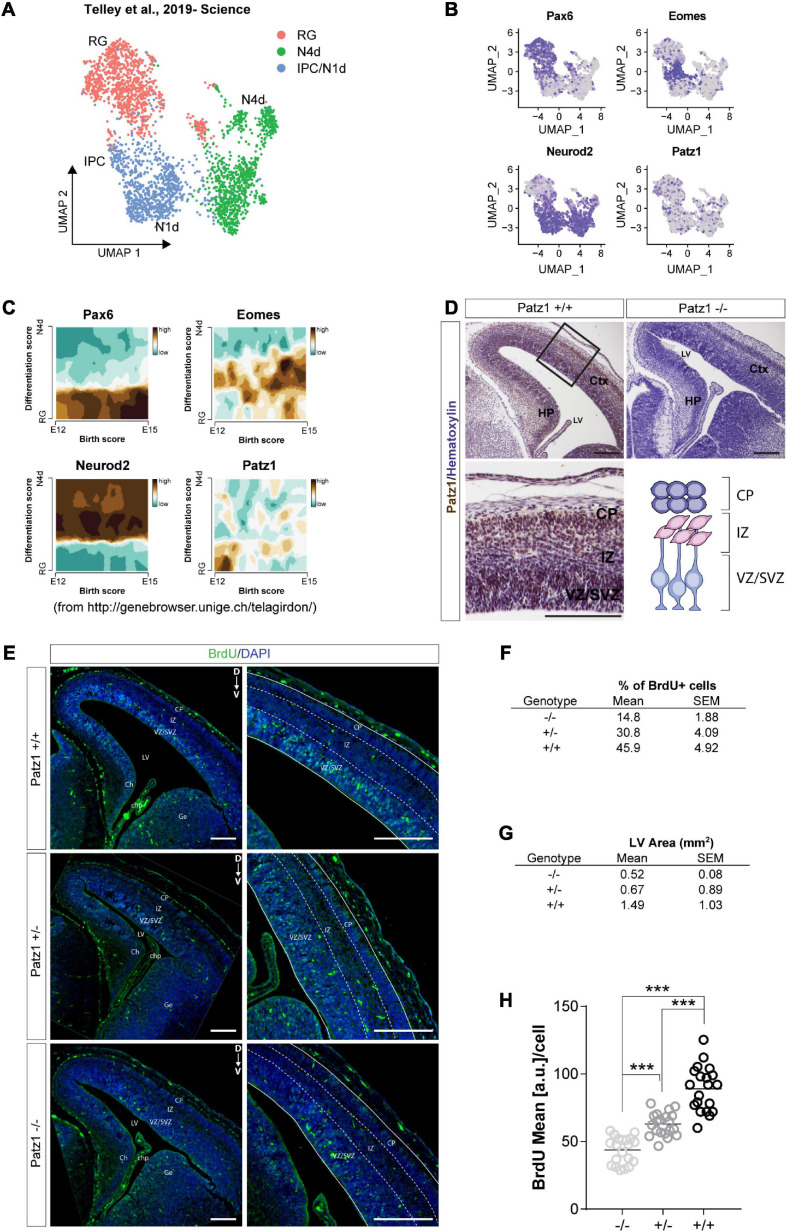
Analysis of *Patz1* expression dynamic in cortical progenitors and incorporated BrdU assay in *Patz1*-knockout embryonic VZ-SVZ. **(A)** UMAP showing cellular composition of the dataset of Telley et al. (2019), including presumptive somato-sensory cortex of E12, E13, E14, and E15 mouse embryos. **(B)** Feature Plots of selected progenitors and neuronal markers. **(C)** Trajectory plots showing the correlation between differentiation score and age (developmental stage) of selected genes. **(D)** Representative images and relative higher magnification of mouse embryonic brain coronal section (E15.5) immunolabeled with Patz1. **(E)** Representative images and relative higher magnification of BrdU immunological detection in brain coronal sections of *wild type* (wt) and *Patz1* mutant E15.5 embryos. Proliferating cells appear as light blue, due to overlapping of blue (DAPI) and green (BrdU) colors. **(F,G)** Tables showing the percentage of BrdU^+^ cells in the VZ/SVZ **(F)** and lateral ventricle area **(G)** according to genotype. **(H)** Dot Plot showing the BrdU mean intensity level per cell in the different genotypes. The significant differences are indicated by asterisks. ^∗∗∗^*P* < 0.001, as assessed by ANOVA followed by Tukey’s multiple comparisons test. Scale bars: 100 μm. RG, radial glia, IPC, intermediate progenitors; N1d, 1 day old Neurons; N4d; 2 days old neurons; CP, Cortical Plate; IZ, intermediate zone; VZ/SVZ; ventricular zone/subventricular zone; LV, lateral ventricle, Ch, cortical hem; Chp; choroid plexus; Ge; ganglionic eminence.

To analyze *Patz1* expression levels across development in different cellular populations we took advantage of different publicly available datasets. Normalized fragments per kilobase of exon model per million reads mapped (FPKM) expression values were downloaded from “Mouse RNA-seq time-series of the development of seven major organs” experiment of EMBL-EBI Expression Atlas (EMBL-EBI Expression Atlas, 2020; Papatheodorou et al., 2020). The experiment comprises gene expression data (mRNAseq) from different tissues (among them heart, brain, liver, testis, ovary) across several time points from embryonic day 10.5 (E10.5) to postnatal day 63 (P63). Expression data relative to *Patz1* in this dataset are represented in the heatmap (A) and in the barplot (B) in Figure 1. Two single cell RNA-seq dataset were also analyzed (©2020 10× Genomics 10K E18 Brain cells; Telley et al., 2019- GSE118953). We downloaded row counts and metadata relative to 10,000 cells from E18 mouse brain from the 10X Chromium website and data relative to the Telley dataset using GSE118953 accession number in the GEO database^1^. Clustering analysis was performed with R version 4.0.3 (2020-10-10) using Seurat package version 3.2.3 (Satija et al., 2015; Butler et al., 2018; Stuart et al., 2019). The dataset was normalized using standard SCT normalization pipeline^2^. A cell cycle score was also assigned to each single cell using the Seurat pipeline but was not used as variable to regress during normalization because considered biologically relevant. We considered the top 60 statistically significant principal components as input for UMAP dimensional reduction, using the DimPlot function. To identify cellular clusters, we used FindClusters function considering 0.2 resolution for both datasets. The robustness of the clustering was validated using an *in silico* downsampling approach. To annotate cell clusters, top 20 differentially expressed genes (markers) per cluster were considered and the most relevant were plotted using DotPlot function to check cell type specificity. Data relative to 10X-Chromium dataset were shown in Figures 2A–D, whereas data relative to the dataset of Telley et al. (2019) were shown in Figures 3A–C. We also analyzed bulk-RNAseq data derived from purified NS/PCs from adult murine SVZ (Belenguer et al., 2021). The normalized expression matrix of the dataset of Belenguer et al. (2021) was downloaded from GEO database using GSE138243 accession number. Expression data relative to *Patz1, Cd9, Slc1a3, Tbr2, Dcxa*, and *Cd24a* genes were reported in the heatmap (scaled data) and in the floating box plot (normalized expression) in Figures 2E,F.

### Animals

*Patz1*-knockout mice were previously described (Valentino et al., 2013b). C57/BL6 *wild type* mice were used to collect mouse brains at different embryonic and post-natal stages of development. Mice were maintained under standardized non-barrier conditions in the Laboratory Animal Facility of Istituto dei Tumori di Napoli (*Patz1*-knockout mice and their *wild type* controls) and in the Animal Facility of the Institute of Genetics and Biophysics (C57/BL6 *wild type* mice showed in Figure 1) in Naples, Italy. All studies were conducted in accordance with the Italian regulations for experimentations on animals (prot. no. 576/10 approved by the Italian Ministry of Health on 3 February 2011).

### *Patz1* Genotype Analysis

Tail pieces from both embryos and adult mice were lysed in tail digestion buffer (50 mM Tris, pH 8.0; 100 mM EDTA, pH 8.0; 100 mM NaCl; 1% SDS) *plus* proteinase K (0.6 mg/ml) over-night at 55°C. Genomic DNA was extracted by phenol/chloroform/isoamyl alcohol (1:1), precipitated with 95% ethanol and dissolved in TE (Tris/EDTA) buffer, pH 7.5. A set of three primers was used to detect both normal and mutant *Patz1* alleles by polymerase chain reactions (PCR), as previously described (Monaco et al., 2018). The primers used were: 5′—GCC TTC TTG ACG AGT TCT TC–3′/5′—CCA CAC CAT CAA AGT TGG–3′ for the knockout allele; 5′—AAG CAA GTG GCT TGT GAG–3′/5′—CCA CAC CAT CAA AGT TGG–3′ for the *wild type* allele.

### Brain Dissection

For embryonic brain collection, C57/BL6 mice were crossed. Noon of the day on which the vaginal plug was detected was considered as 0.5 days *post coitum* (dpc) in the timing of the embryo collection. Timed pregnant females were sacrificed by cervical dislocation and embryos were dissected from decidual tissue in cold PBS. For each embryonic stage two different embryonic litters were analyzed. For each litter, brains from three embryos were dissected into the different territories. Corresponding brain territories of the same litter were pulled together to increase starting material. At each postnatal stage, two mice were sacrificed by cervical dislocation and brains were extracted and then dissected into the different territories. Each postnatal brain territory was analyzed separately.

The dissection was started separating the head from the body using scissors, then, a midline incision along the integument from the neck to the nose was made to expose the skull or its primordium. Any residual tissue was removed using scissors or tweezers. Before to proceed to surgery, all the dissection instruments were disinfected with 70% ethanol. Moreover, the instruments were sterilized with 70% ethanol before the removal of each tissue in order to avoid contaminations.

### RNA Extraction and Reverse Transcription–Quantitative Polimerase Chain Reaction (RT-qPCR)

Five hundred microliter of Trizol reagent (Sigma, St. Louis, MO, United States) were added to each sample, except for adult cerebellum and mesencephalon in which 1 ml was added, for subsequent RNA extraction according to the manufacturer’s protocol. Recovered RNA was reverse transcribed using M-MuLV Reverse Transcriptase (Roche Diagnostics). qPCR was performed with SYBR Green PCR Master Mix (Life Technologies) under the following conditions: 10 min at 95°C, followed by 40 cycles (15 s at 95°C and 1 min at 60°C). Each reaction was performed in duplicate in at least three independent experiments. The 2^–ΔΔ*Ct*^ method (Livak and Schmittgen, 2001) to calculate the relative expression levels (fold change) was used. Amplification of two housekeeping genes, *Glucose-6-Phospate Dehydrogenase* (*G6PD*) and *Ribosomal Protein S9* (*RPS9*) genes, was used to normalize the amount of cDNA. For analysis of brain territories in Figure 1, E11.5 or E14.5 samples have been used alternatively as calibrators to calculate the relative expression. For analysis of neurospheres, both proliferating and differentiated in Figure 6, one of control samples, randomly chosen and different for each independent experiment, was used as calibrator to calculate the ΔΔCt. Sequences for forward (Fw) and reverse (Rv) primers were as follows: *Patz1* Fw = 5′-GAG CTT CCC CGA GCT CAT-3′; *Patz1* Rv = 5′–CAG ATC TCG ATG ACC GAC CT–3′; *Nanog* Fw = 5′–GCC TCC AGC AGA TGC AAG–3′; *Nanog* Rv = 5′-GGT TTT GAA ACC AGG TCT TAA CC-3′; *Nestin* Fw = 5′—GAG AAG ACA GTG AGG CAG ATG AGT T–3′; *Nestin* Rv = 5′—GCC TCT GTT CTC CAG CTT GCT–3′; *Tubb3* Fw = 5′—GAA TGA CCT GGT GTC CGA GT–3′; *Tubb3* Rv = 5′—CCG ATT CCT CGT CAT CAT CT–3′; *G6pd* Fw = 5′—CAG CGG CAA CTA AAC TCA GA–3′; *G6pd* Rv = 5′—TTC CCT CAG GAT CCC ACA C—3′; *Rps9* Fw = 5′—CTG GAC GAG GGC AAG ATG AAG c—3′; *Rps9* Rv = 5′—TGA CGT TGG CGG ATG AGC ACA—3′.

### Bromodeoxyuridine Pulse-Labeling and Detection

Heterozygous *Patz1*-mutant mice were mated and vaginal plug monitored each day to establish the coitum day. At 15.5 days dpc pregnant mice received a single intra-peritoneal injection of 5′-bromo-2′-deoxyuridine (BrdU) (50 mg/kg) and, 2 h later, they were euthanized. One embryo for each genotype was extracted, post-fixed in 4% paraformaldehyde for 24 h and then kept in 70% ethanol at 4°C before to be processed for immunofluorescence analysis. Embryos were then dehydrated through ascending ethanol, washed in Toluene and Toluene-Paraffin 1:1, included in paraffin and then sectioned using a microtome to undergo histological analyses (Giorgio et al., 2014). For details see elsewhere (Q4Lab, 2016). Briefly, cutting angle and thickness have been set, respectively, at 5 and 8 μm. Paraffin sections have been dewaxed in xylene (pre-warmed at 55–65°C) twice for 5 min and then rehydrated through sequential steps in gradually less concentrated ethanol solutions and rinsed in tap water, at the end. Slides have been heated in a 10 mM sodium citrate pH 6.0 in a microwave to expose the antigens (Pisapia et al., 2020). Tissue sections have been incubated 1 h at R.T. with a solution 1% milk, 10% FBS, 1% BSA, 1X Sodium Azide, 0,5% Tween in PBS 1X and then O.N. at 4°C with a rat-monoclonal BrdU antibody (Novus Biologicals NB500-169) at dilution 1:400. Sections have been rinsed and incubated 1,5 h at R.T. with a Donkey α-rat Alexa Fluor- 488 secondary antibody (Molecular Probes A-21208) at 1:400 dilution, counterstained with Hoechst 1:5,000 and then mounted.

### *Patz1* Immunological Detection

Sections were dewaxed in xylene, hydrated in graded series of alcohol and subjected to heat-induced antigen retrieval (Guadagno et al., 2017). After blocking endogenous peroxidase activity, the tissue was incubated for 1 h at R.T. with the blocking solution previous described and then with our previously characterized polyclonal antibody able to recognize both human and mouse Patz1 proteins (Valentino et al., 2013a) at a dilution 1:40 (V/V) O.N. Subsequently, the slices were rinsed and incubated with the biotinylated secondary antibody, at room temperature, for 30 min. The bound antibody complexes were stained for 3–5 min with diaminobenzidine, and slides were then counterstained with hematoxylin (30 s), dehydrated and mounted.

### Neurosphere Culture, Proliferation, and Differentiation Assays

Before surgery all the dissection instruments have been disinfected with 70% ethanol. Adult mice or pregnant females were euthanized. SVZ dissection from 4 *wild type*, 3 *Patz1^+/–^* and 2 *Patz1^–/–^* adult mice or total forebrain dissection from 6 *wild type*, 6 *Patz1^+/–^* and 2 *Patz1^–/–^* 13.5 dpc embryos was carried out under stereoscopic control in ice-cold disodium phosphate buffer solution supplemented with glucose 0.6% (PBSg). The dissected samples were transferred into a new sterile cold PBSg buffer and brought under the hood where they were mechanically dissociated in single cell suspension.

For each sample, cells were counted and plated at the density of 3,000 cells/cm^2^ in 14–16 ml of serum-free medium comprising 1:1 mixture of DMEM and F12 supplemented with 2 mM glutamax, N2 supplement (1:100), B27 minus Vitamin A (1:50) (Invitrogen), 20 ng/ml EGF (Sigma), 20 ng/ml bFGF (Sigma) and penicillin/streptomycin (1:100), into T75 neurosphere-flasks in a prewarmed culture medium, to obtain a single embryo cell culture. 1 × 10^5^ and all cells recovered from dissection were plated for embryonic and adult neurospheres, respectively. The number of neurospheres/brain were counted under microscope after 4 days in culture for embryonic cells and 7 days for adult ones, and their diameter measured by ImageJ. Only neurospheres > 40 μm in diameter were considered (Scopa et al., 2020). Primary neurospheres (P0) were then disaggregated by incubating with TripleExpress dissociation reagent (Invitrogen) for 5 min at room temperature. After the incubation, the dissociation reagent was aspirated, the neurosphere were mechanically dissociated and replated (1 × 10^5^/plate) to form secondary neurospheres (P1). Initial self-renewal activity was evaluated by counting the number of P1 neurospheres/cell seeded ^∗^ 100.

For differentiation assay, neurospheres at passage 3 were dissociated to obtain single cell suspension, as described above and plated on poly-D-lysine coated multiwell plates at a density of 50,000 cells/cm^2^. Cells were grown in absence of growth factors for 6 days in a 1:1 mixture of Neurobasal and DMEM/F12 medium supplemented with B27 plus Vitamin A (1:50), 2 mM glutamax (Invitrogen) and penicillin/streptomycin (1:100). Half of the differentiation medium was replaced every other day.

### Image Quantification

ImageJ/Fiji software was used to perform image analysis (Rueden et al., 2017). To compute the percent of BrdU^+^ cells (Figure 3F), 10 different images were analyzed per sample by defining the number of BrdU^+^ cells over Hoecsht^+^ cells on 5 different regions of interest (ROIs) manually set on VZ/SVZ using Cell Counter ImageJ Plug-in. The lateral ventricle area was computed over 5 different images per sample spanning from anterior to posterior regions, by segmenting Hoecsht signal and automatically defining ROIs over segmentation using ImageJ default threshold algorithm (Figure 3G). BrdU intensity signal per cell was computed by segmenting single positive nuclei of 3 different images per sample using ImageJ MaximalEntropy threshold function and computing the Mean Intensity arbitrary unit (a.u.) score using the Analyze Particle function. Representative quantifications of this analysis are shown in the dot plot in Figure 3H.

To compute the percent of β-III-tubulin^+^ cells (Figure 6F), 5 different images were analyzed per sample by defining the number of β-III-tubulin^+^ cells over Hoecsht^+^ cells using Cell Counter ImageJ Plug-in. NeuroJ Plung-in was used to perform neurite guided tracing and perform number (Figure 6H), length (Figure 6I), and fluorescence score (Figure 6J) analysis of 3–5 neurons per image for at least 3 images per sample.

### Statistical Analysis

Pearson correlation test was performed among the *Patz1* expression levels detected and the age of the analyzed samples (Figure 1) and between *Patz1* and *Nanog* expression in *Patz1^+/+^* and *Patz1^+/–^* adult neurospheres (Supplementary Figure 1). Ordinary one-way analysis of variance (ANOVA) followed by Tukey’s multiple comparisons test was applied for comparison of all the other sets of data. The values analyzed are the average ± SD of at least two biological replicates and three independent experiments. All tests were assessed using GraphPad Prism 7 software, La Jolla (CA), United States. Statistical significance was indicated by ^∗^*p* < 0.05; ^∗∗^*p* < 0.01; ^∗∗∗^*p* < 0.001; ^****^p < 0.0001 vs. *wild type* control when not differently specified in the figures by linking lines.

## Results

### *Patz1* Is Expressed During Mouse Brain Development and Turns Down in Adult Brain

To get insight into *Patz1* expression pattern during embryonic development, we took advantage of the Expression Atlas publicly available dataset containing RNA-seq data from different tissues across multiple timepoints during both embryonic and postnatal development (Figures 1A,B). As shown in Figure 1A, *Patz1* expression is frequently higher during embryogenesis than in postnatal and adult stages, with the strongest expression of *Patz1* in embryonic brain respect to the other tissues analyzed (heart, liver, ovary, and testis). Interestingly, during brain development, a decreasing trend of *Patz1* expression with respect to time, from E10.5 to P63, is quite evident (Figure 1B).

To confirm and expand these findings, we analyzed, by means of qPCR, *Patz1* transcript distribution levels in specific mouse brain territories at different time points during both embryonic (11.5, 14.5, and 17.5 dpc) and postnatal (14 days and adults) life. The results are shown in Figure 1C and confirm the existence of a temporal *Patz1* expression gradient, with higher levels of expression at early embryonic stages, that decrease during embryogenesis and even more during postnatal (P14) and adult life, when *Patz1* expression reaches the minimum measured level. This trend is detectable in the whole brain (Pearson correlation index = −0.6249; *p* = 0.0014) and also in the specific structures analyzed, as reported in Figures 1C–E. Conversely, there is no preferential spatial distribution at the different embryo and adult stages. Interestingly, looking at the relative expression of *Patz1* in the adult, a higher expression level was detected in telencephalic structures (olfactory bulbs, cortex, and hippocampus), in which neurogenesis mainly occurs after birth, respect to the most posterior ones (Figure 1C).

### Patz1 Is Enriched in Proliferating Neural Progenitor Cells Inside the SVZ and Appears Involved in Their Maintenance

Given the complex cellular heterogeneity and temporal dynamics of embryonic and adult brain tissue, we took advantage of publicly available single-cell and bulk RNA sequencing datasets to get insight into *Patz1* expression level in neural progenitors during brain development. By analyzing the 10× Genomics 10K E18 Brain cells dataset, we found an enrichment of *Patz1* expression in early apical radial glia/neural stem cells (RG/NSCs) and intermediate progenitors (IPCs) (Figures 2A–D). We also examined *Patz1* expression in a dataset of bulk RNA sequencing from sorted types of progenitors from mouse adult SVZ (Belenguer et al., 2021), finding that *Patz1* was expressed in the entire proliferating lineage with an enrichment in neural progenitor cells (NPCs) and neuroblasts (NBs) (Figures 2E,F). A further analysis, on single-cell RNA sequencing of sorted progenitor cells belonging to presumptive somato-sensory cortex of E12, E13, E14, and E15 mouse embryos (Telley et al., 2019), confirmed an enrichment of *Patz1* expression during development in early apical radial glia/neural stem cells (RG/NSCs), mainly at the E12 stage (Figures 3A–C). Accordingly, Patz1 immunodetection at E15.5 showed a strong immunoreactivity in the cortical plate as well as in the VZ/SVZ of *wild type* embryo (Figure 3D). As expected, no Patz1 immunosignal was detected in corresponding brain sections of null mutants (Figure 3D). All these data point out to a possible function of Patz1 during embryonic neurogenesis. Then, to have a first indication of the impact of Patz1 depletion on proliferating cells in the periventricular area, we examined the *in vivo* proliferation inside the SVZ of 15.5 dpc *Patz1*-null and heterozygous mouse embryos, compared to *wild type* control, using a saturation BrdU (5′-bromo-2′-deoxyuridine) pulse-labeling method (Kuhn et al., 1996; Hayes and Nowakowski, 2002) that could label the entire pool of proliferating NS/PCs within a 12 h-period (Figure 3E). BrdU is a thymidine analog that incorporates into dividing cells during DNA synthesis. BrdU-labeling appears weaker and unevenly distributed in the SVZ of *Patz1*-null and heterozygous mutants compared to control brains. Quantitative analysis indicates a reduction of the number of proliferating cells (Figure 3F) and a significant decrease of BrdU incorporation level (Figure 3H) coupled to an overall reduction of lateral ventricle area (Figure 3G) in both *Patz1* mutants compared with control. Interestingly, the heterozygous shows mean values lower than *wild type* and higher than *Patz1* null mutant for all parameters analyzed.

### Reduced Proliferative Capacity in Patz1-Knockout-Derived Neurospheres

**FIGURE 4:**
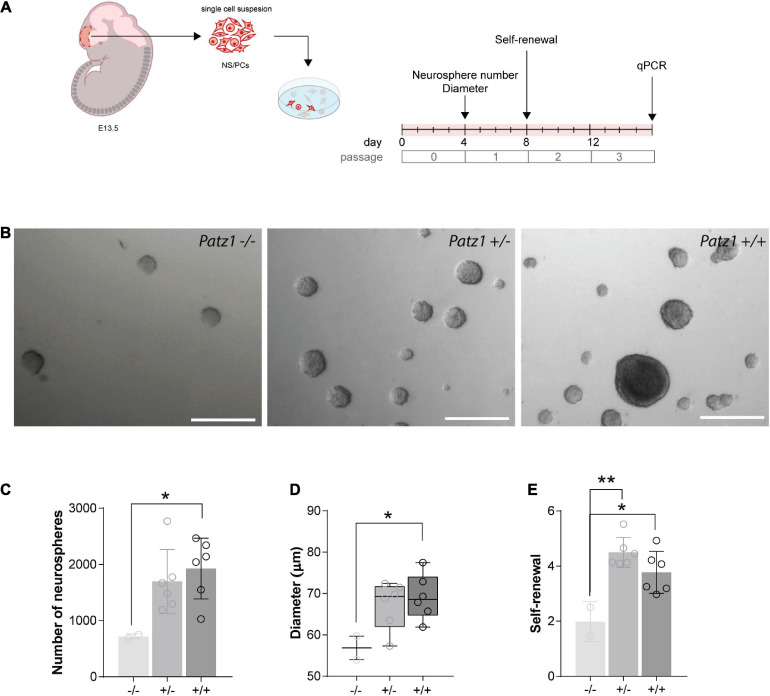
*Ex vivo* proliferation assay of 13.5 dpc embryonic telencephalon-derived neurospheres. **(A)** Experimental scheme showing the brain region microdissected to produce neurospheres, and relative experiment timeline. **(B)** Representative images of neurospheres from *Patz1*^–/–^, *Patz1^+/–^* and *wild type* embryos. Scale bars = 100 μm. **(C)** Bar/dot plot (means ± SD) showing the number of primary neurospheres (P0) obtained by plating 1 × 10^5^ cells/embryo obtained from the dissected tissue. **(D)** Box dot/plot showing the diameter of P0 neurospheres (median ± min to max). **(E)** Neurosphere self-renewal capacity (number of neurospheres of the first-generation (P1)/plated cells * 100). All the significant differences are indicated by asterisks. *P < 0.05; **P < 0.01, as assessed by ANOVA followed by Tukey’s multiple comparisons test.

**FIGURE 5:**
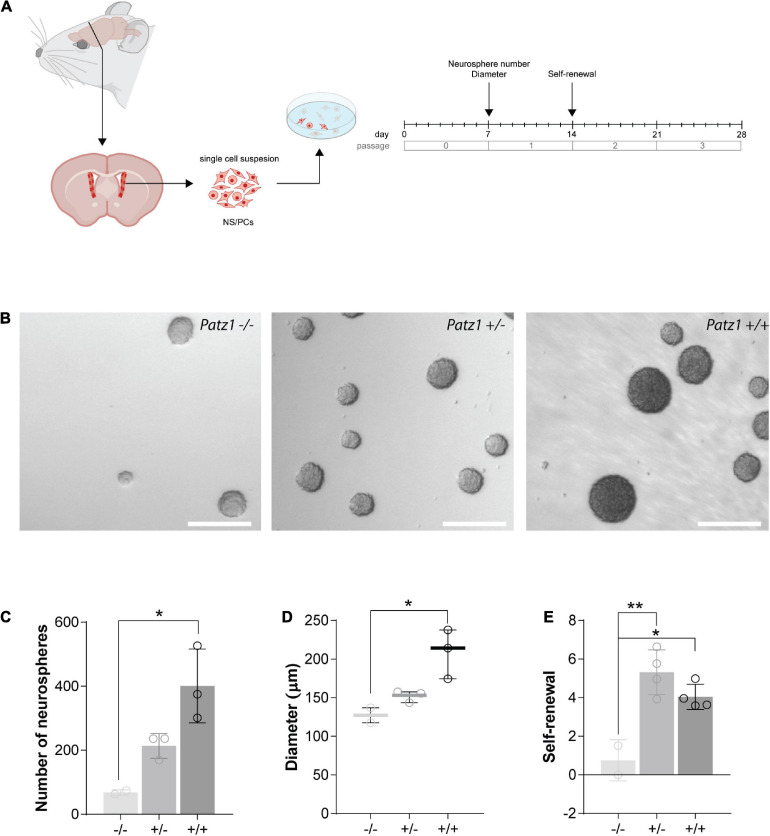
*Ex vivo* proliferation assay of SVZ-derived NS/PCs from adult mice. **(A)** Experimental scheme showing the brain region microdissected to produce neurospheres and relative experiment timeline. **(B)** Representative images of neurospheres from *Patz1*^–/–^, *Patz1^+/–^* and *wild type* mice. Scale bars = 100 μm. **(C)** Bar/dot plot (means ± SD) showing the number of primary neurospheres (P0) obtained from the adult brains by plating all the cells recovered by SVZ dissection. **(D)** Box dot/plot showing the diameter of the P0 neurospheres (median ± min to max). **(E)** Neurosphere self-renewal capacity (number of neurospheres of the first-generation (P1)/plated cells * 100). All the significant differences are indicated by asterisks. *P < 0.05; **P < 0.01, as assessed by ANOVA followed by Tukey’s multiple comparisons test.

To effectively measure the number of NS/PCs and their proliferation rate, we performed neurosphere assays on both *Patz1* mutant and *wild type* brains. In this assay NS/PCs derived from SVZ of *Patz1*^–/–^, *Patz1^+/–^* and *wild type* adult mice (more than 1 month up to 21 months of age) as well as from 13.5 dpc embryonic telencephalon (from which SVZ arises), were allowed to proliferate in culture at very low density to form spheroid cell aggregates called neurospheres. The number of neurospheres was determined after 4 days in culture for embryonic and 7 days for adult neurospheres (Figures 4A, 5A). Under these conditions, primary neurosphere colonies are derived from single cells and can be used as a good model of the number of *in vivo* NS/PCs (Morshead et al., 2003; Coles-Takabe et al., 2008). In agreement with *in vivo* BrdU labeling data (Figures 3E–H), the number of primary neurospheres (P0) isolated from *Patz1*^–/–^ embryonic telencephalons was significantly lower than *wild type* counterparts (mean difference −1208; 95% CI of diff. −2377 to −39.62) (Figures 4B,C). The diameter of each neurosphere, an indirect measure of the ability of cells to grow and proliferate, was also determined, showing a significant reduction in neurospheres derived from *Patz1*^–/–^ embryos compared to the *wild type* (mean difference −12,36; 95% CI of diff. −24.48 to −0.2338) (Figure 4D). Furthermore, to assess the initial NS/PC self-renewal capability, primary neurospheres were dissociated into single cells after 4–7 days in culture and 10,000 cells per sample were cultured as above to form secondary neurospheres (P1) that were counted (day 8 and 14 for embryonic and adult neurospheres, respectively). As shown in Figure 4E, we observed a significant reduction (mean difference = −1.787; 95% CI of diff. −3.261 to −0.3126) in the number of P1 neurospheres from *Patz1*^–/–^ embryos compared to *wild type* controls, indicating the impairment of the self-renewal ability of *Patz1*-null NS/PCs. The reduction of the number (mean difference −332.9; 95% CI of diff−561.5 to −104.4), the diameter (mean difference −81.52; 95% CI of diff. −145.8 to −17.26) and the self-renewal capability (mean difference −3.425; 95% CI of diff. −6.053 to −0.7973) of NS/PCs lacking Patz1 was also evident in the SVZ of adult brain (Figure 5). Even though no significant difference was observed in the number and size of neurospheres from the heterozygous *Patz1^+/–^* brains, compared to both *Patz1*^–/–^ and *wild type* samples, data from both embryonic and adult samples suggest for *Patz1^+/–^* heterozygotes an intermediate trend between *Patz1*-null mutants and *wild type* controls (Figures 4C,D, 5C,D). Noteworthy, both embryonic and adult *Patz1^+/–^* NS/PCs showed a self-renewal capability significantly higher than *Patz1*-null homozygotes and comparable to *wild type* (Figures 4E, 5E).

Overall, these data provide evidence for a role of Patz1 in the maintenance, proliferation and self-renewal capability of NS/PCs at both embryonic and adult stages.

### Downregulation of Stem Cell Related Genes and Enhanced Neuronal Differentiation in *Patz1*-Knockout-Derived Neurospheres

**FIGURE 6:**
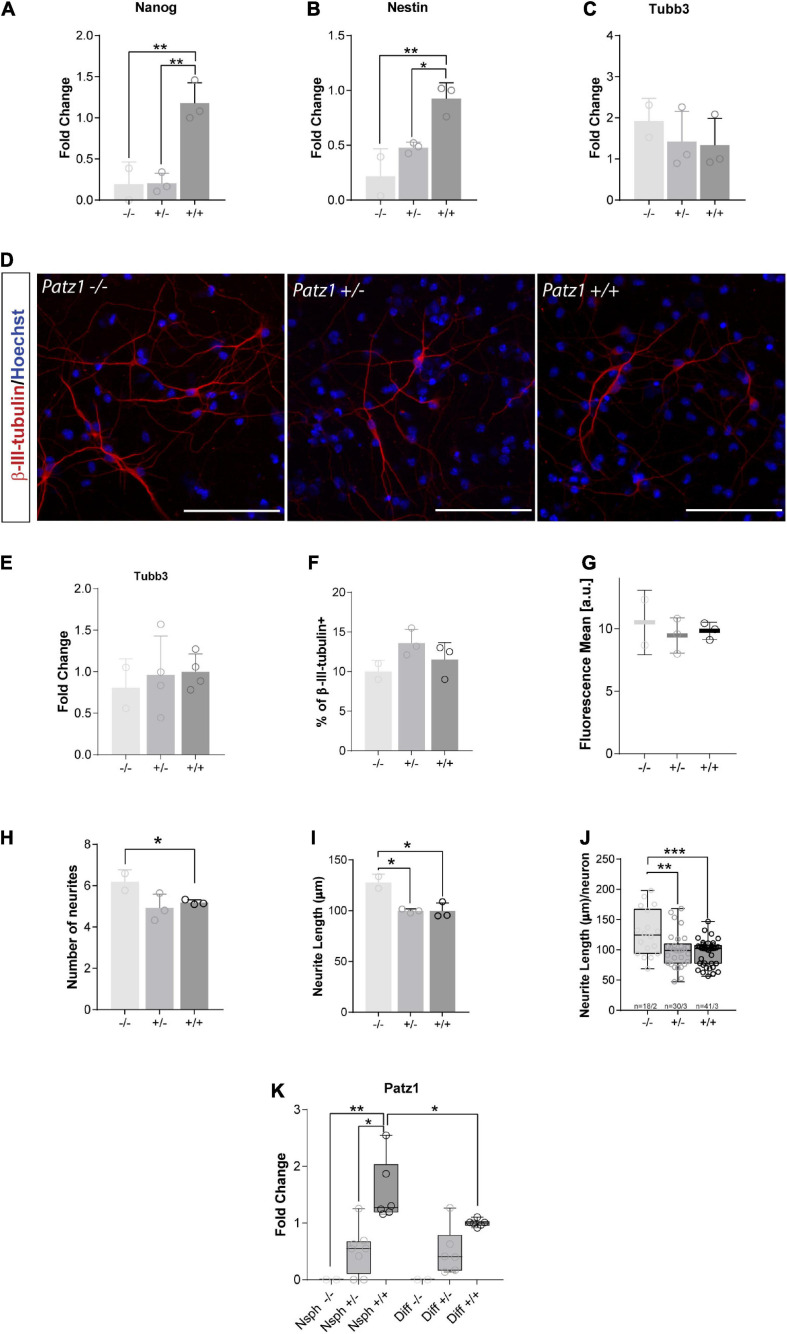
Real time analysis of gene expression and neuronal differentiation in proliferating neurospheres and differentiated cells. **(A–C)** Relative expression of the genes indicated at the top of each graph is expressed as fold changes with respect to the level of a *wild type* control, randomly chosen and different for each independent experiment. Mean values ± SD of at least three independent experiments performed in duplicate on samples derived by 2 *Patz1*^–/–^, 3 *Patz1*^+/–^ and 3 *wild type* embryo-derived neurospheres are reported. **(D)** Representative images of differentiated cells immunolabelled with β-III-tubulin neuronal marker. **(E)** Relative expression of *Tubb3* in differentiated cells as assessed by RT-qPCR. **(F–J)** Quantitative analysis of the experiment shown in D plotted to show the percentage of β-III-tubulin^+^ cells (neurons) **(F)**, the mean fluorescence of β-III-tubulin intensity **(G)**, the number **(H)** and length **(I)** of neurite branches, and the neurite length/neuron ratio **(J)**. **(K)** Relative expression level of *Patz1* in proliferating neurospheres (Nsph) and after 6 days in differentiation medium (Diff). *p < 0.05; **p < 0.01; ***p < 0.001 vs. *wild type* control, if not diversely indicated by connecting line, as assessed by ANOVA followed by Tukey’s multiple comparisons test.

RNA extracted from embryo-derived neurospheres, after at least three passages in culture (Figure 4A), under proliferating conditions, was used to analyze expression of genes characteristic of stemness (*Nanog*) and neural progenitor (*Nestin*) phenotype in proliferating neurospheres, or neuronal (*Tubb3*) cells. Nanog is a DNA binding homeobox transcription factor involved in embryonic stem cell proliferation, renewal, and pluripotency, which is a general marker of stemness; Nestin, neuroepithelial stem cell protein, is an intermediate filament protein that has generally been considered to be a marker of NS/PCs and is crucial for their self-renewal and survival; while *Tubb3*, coding for β-III-tubulin, correlates with neuronal differentiation (Zhang and Jiao, 2015). In agreement with the above data, both *Nanog* and *Nestin* genes were significantly downregulated in homozygous *Patz1*-null NS/PCs with respect to *wild type* (Figures 6A,B), confirming also at molecular level that *Patz1* inactivation strongly affects the stem/progenitor phenotype. Moreover, *Patz1*-null heterozygous neurospheres also showed a significant downregulation of *Nanog* and *Nestin* expression with respect to the control ones. However, *Nestin* expression levels of *Patz1^+/–^* NS/PCs settle in the middle between *Patz1^–/–^* and *wild type* NS/PCs, whereas *Nanog* expression of *Patz1^+/–^* NS/PCs was as low as in the homozygous *Patz1*-null neurospheres. These results might suggest a significant reduction of the Nestin^+^ and Nanog^+^ cells in Patz1-knockout NS/PCs compared to *wild type* controls. However, according to the role of Patz1 as a positive regulator of Nanog expression in mouse embryonic stem cells (Ow et al., 2014), Patz1 might activate *Nanog* expression also in NSCs and the reduced levels of Nanog could be partly due to the direct modulation of Nanog promoter by Patz1. Consistently, *Patz1* and *Nanog* gene expression were highly correlated in adult neurospheres as well (Pearson correlation index = 0.9; *p* = 0.0069) (Supplementary Figure 1).

Conversely, the expression of *Tubb3* did not change significantly in the three groups (Figure 6C), even though a slight increasing trend was visible among the different genotypes from *Patz1^+/+^* to *Patz1*^–/–^ proliferating neurospheres, which might suggest an accelerated differentiation into neurons of the *Patz1*-null neurospheres. To verify this hypothesis, neurospheres were cultured for 6 days in differentiating conditions and neuronal differentiation was confirmed by β-III-tubulin immunodetection (Figure 6D). *Tubb3* transcript levels after neurospheres differentiation did not show any significant difference among the different genotypes (Figure 6E), as well as the number of β-III-tubulin^+^ cells detected (Figure 6F) and the mean fluorescence level per neuron (Figure 6G). However, both number and length of neurites were higher in *Patz1*^–/^*^–^* than in both *Patz1^+/–^* and *Patz1*^+/+^ cells (Figures 6H–J), indicating that *Patz1*-null neurospheres can go toward an enhanced neuronal differentiation compared to control. Consistent with the genotype, *Patz1* expression in heterozygous was about half the level of *wild type* cells, while it was not detected at all in *Patz1*-null samples (Figure 6K). Interestingly, its expression was lower in differentiated than in proliferating cells, once again supporting a role of Patz1 in counteracting neuronal differentiation.

## Discussion

Growing evidences suggest that Patz1 and stemness are closely associated. *Patz1* is required for pluripotency maintenance of mouse embryonic stem cells (ESCs), by regulating expression of pluripotency master genes *Pou5f1* and *Nanog* (Ow et al., 2014), and its gene dosage is crucial for an efficient reprogramming of mouse induced pluripotent stem (iPS) cells (Ma et al., 2014). Moreover, during mouse embryonic development, *Patz1* gene expression has been detected in actively proliferating neuroblasts of the periventricular and subventricular neocortical neuroepithelium, and *Patz1^–/–^* embryos showed anatomical reduction of the SVZ (Valentino et al., 2013b).

Here, we confirmed and expanded these previous results, revealing a temporal decreasing expression gradient of *Patz1* gene during mouse brain development. Indeed, *Patz1* transcription levels in the brain decrease during embryogenesis and even more at postnatal stages, reaching the minimum levels in adult mice. In adults, higher *Patz1* expression levels correspond to brain structures characterized by still active neurogenesis, such as olfactory bulbs, cortex and hippocampus. The maintenance of high *Patz1* expression in the adult hippocampus, a region that is essential for many forms of learning, memory and mood regulation (Christian et al., 2014), suggests that aberrant expression of *PATZ1* could contribute to neurological and psychiatric disorders. Indeed, adult-generated neurons and the dynamic regulation of neurogenesis by epigenetic factors are relevant for neuropsychiatric disorders, including depression, addiction, and epilepsy (Hsieh and Eisch, 2010). Consistently, *PATZ1* has been identified as one of the differentially regulated key genes in a form of depression (Xu et al., 2015). Moreover, very recently, PATZ1 has also been associated to Parkinson’s disease, consistent with its possible contribution to neurodegenerative diseases (Schilder and Raj, 2020). However, no studies have so far specifically investigated the role of *PATZ1* in NS/PCs.

We now showed an *in vivo* analysis of BrdU incorporation in embryonic brain of Patz1-null mutant mice, confirming previous observations (Valentino et al., 2013b) and suggesting a different distribution and lowered number of proliferating cells inside the SVZ of *Patz1*-null mutants compared to *wild type* embryos. Quantitative and qualitative analysis of neurospheres in culture is extremely helpful in studying both self-renewal and differentiation potential of NS/PCs and dissecting mouse mutant neural phenotype (Morshead et al., 2003; Coles-Takabe et al., 2008; Liguori et al., 2009). By using this approach on our *Patz1*-knockout mouse model, we explored the role of Patz1 in self-renewal and proliferation of NS/PCs. Albeit with the limitation of a small number of Patz1^–/–^ samples, due to the high and early embryonic lethality of this genotype in our current mouse colony, the data indicate that Patz1 contributes to proliferation and self-renewal of NS/PCs inside the SVZ, likely playing a role in the early development of the mouse brain, and lately in the maintenance of the adult neurogenic niches. Indeed, evidence for a role of Patz1 in the maintenance and proliferation of NS/PCs was corroborated by impairment of the self-renewal of *Patz1*-null NS/PCs in both embryonic and adult *ex vivo* neurosphere cultures derived from telencephalon and SVZ, respectively. Consistent with neurosphere functional data, the gene expression analysis in proliferating neurospheres also suggests that *Patz1* inactivation might strongly impair the ability of these NS/PCs to maintain a stemness phenotype. Indeed, the *Patz1*-null neurospheres express significantly lower levels of stemness and neural progenitor markers, such as *Nanog* and *Nestin*, respectively, while the neuronal marker *Tubb3* does not seem to be affected, indicating a specific role of Patz1 in their proliferation and self-renewal. These data are consistent with recent results in other cell types, such as rat thyroid cancer stem-like cells, in which *Patz1* expression enhances self-renewal ability (Vitiello et al., 2019).

We also provided a first evidence of a negative role for Patz1 in the differentiation of embryonic neurospheres, showing enhanced neuronal differentiation in *Patz1*^–^*^/^*^–^ compared to *Patz1^+/–^* and *Patz1^+/+^* cells. Moreover, differentiated *wild type* cells had significantly lower levels of *Patz1* than non-differentiated neurospheres, supporting its opposing role in neurosphere differentiation. Additional neurosphere differentiation experiments are underway to investigate possible additional roles of Patz1 in neuro-glial differentiation. Consistently, a recent study linked PATZ1 to neuron and glia differentiation through its interaction with the variant polycomb repressor complex 1 component PCGF1, which also interacts with NANOG and OCT4, and contribute to the maintenance of the undifferentiated phenotype of NT2 teratocarcinoma cell line that, upon treatment with all-*trans* retinoic acid, can differentiate to both neuron and glia (Oliviero et al., 2015). Moreover, it has recently been shown that during neural differentiation, *Nanog* prevents the upregulation of genes important for neural specification (Barral et al., 2019). As a further support, our previous studies have associated PATZ1 to cell plasticity, being it involved in both epithelial-mesenchymal and proneural-mesenchymal transition (Chiappetta et al., 2015; Fedele et al., 2019).

It is well known that epigenetic mechanisms, including DNA and histone modifications, play critical roles in different stages of neurogenesis, and that aberrant epigenetic regulation also contributes to the pathogenesis of various brain disorders (Yao et al., 2016). PATZ1 belongs to a large family of transcription factors, named POZ-ZF or POK, including BCL6, PLZF, TAZ1, and others, in which the POZ/BTB domain mediates protein-protein interactions, allowing the recruitment of histone deacetylases through co-repressor complexes (Costoya, 2007). PATZ1 is indeed an epigenetic regulator acting through the recruitment of repressor complexes that modulate histone deacetylase levels to the promoter of different genes (Sakaguchi et al., 2010; Cho et al., 2012). Based on these findings, PATZ1 may be one of the key epigenetic factors that, by remodeling chromatin through the recruitment of histone deacetylases, is implicated in neurogenesis and, consequently, neurodegenerative and neuropsychiatric disorders. In this frame, the relatively high expression of PATZ1 in the hippocampus is particularly significant, where adult NSCs generate granular cells that confer the plasticity necessary for memory and behavior (Jessberger et al., 2009; Sahay et al., 2011). PATZ1 in these cells could be crucial for such plasticity, accounting for its involvement in depression (Xu et al., 2015). Indeed, hippocampus is an important anatomical area associated with depression and studies have shown that some antidepressant can treat depression by changing the plasticity of the hippocampus (Xu et al., 2020). Future experiments are needed to verify such hypothesis.

A tight link exists between NSCs and glioblastoma stem cells (GSCs). Glioblastoma (GBM) is the most frequent form of brain tumor in adults and is associated with a poor prognosis in both adults and children due to the poor tumor response to the limited available therapeutic options (Sturm et al., 2014). Various studies have suggested that NSCs might be the cells of origin of GBM, which arises from migration of mutated astrocyte-like NSCs (Alcantara Llaguno et al., 2009; Lee et al., 2018; Altmann et al., 2019). We have previously shown that PATZ1 is overexpressed in both adult and pediatric GBM compared to normal glial cells (Guadagno et al., 2017; Passariello et al., 2019), and that it is specifically expressed in GSCs, where it is associated with the proneural phenotype (Guadagno et al., 2017). Based on the results emerged from the present study, we could hypothesize that PATZ1 overexpression may contribute in maintaining the self-renewal and proliferative capacity of GSCs, likely favoring symmetric over asymmetric stem cell division. It is in fact known that both HMGA1 and p53, two PATZ1 interactors (Fedele et al., 2012; Valentino et al., 2013a; Keskin et al., 2015), are deeply involved in the balance between symmetric and asymmetric stem cell division, which plays a key role in the expansion of the stem cell pool in both NSCs and GSCs (Cicalese et al., 2009; Puca et al., 2019). Indeed, an undue increase in NSC symmetrical divisions, due to the disruption of molecular regulators, is the prelude to the conversion to GSCs and tumors in invertebrates (Caussinus and Gonzale, 2005). Future molecular experiments are needed to clarify this possibility.

## Data Availability Statement

The datasets presented in this study can be found in online repositories. The names of the repository/repositories and accession number(s) can be found in the article/Supplementary Material.

## Ethics Statement

The animal study was reviewed and approved by the Italian Ministry of Health (prot. no. 576/10 approved on 3 February, 2011). Written informed consent was obtained from the owners for the participation of their animals in this study.

## Author Contributions

MF: conceptualization, project administration, and original draft preparation. GLL and SM: methodology. SM, MV, and MD: validation. GLL and MF: formal analysis and supervision. SM, MV, MD, FM, GP, and AL: investigation. CA, GLL, and MF: resources. SM, GLL, and MF: data curation. SM, GLL, LC, and MF: writing, review and editing. LC and GLL: funding acquisition. All authors have read and agreed to the published version of the manuscript.

## Conflict of Interest

The authors declare that the research was conducted in the absence of any commercial or financial relationships that could be construed as a potential conflict of interest.

## Footnotes

1 https://www.ncbi.nlm.nih.gov/geo/query/acc.cgi?acc=GSE118953

2 https://github.com/hbctraining/scRNA-seq
