# Supplementary figures and images for: The Transcription Regulator Patz1 Is Essential for Neural Stem Cell Maintenance and Proliferation
Source: Front Cell Dev Biol. 2021 Apr 7;9:657149. doi: 10.3389/fcell.2021.657149 (PMC8058466; doi:10.3389/fcell.2021.657149)

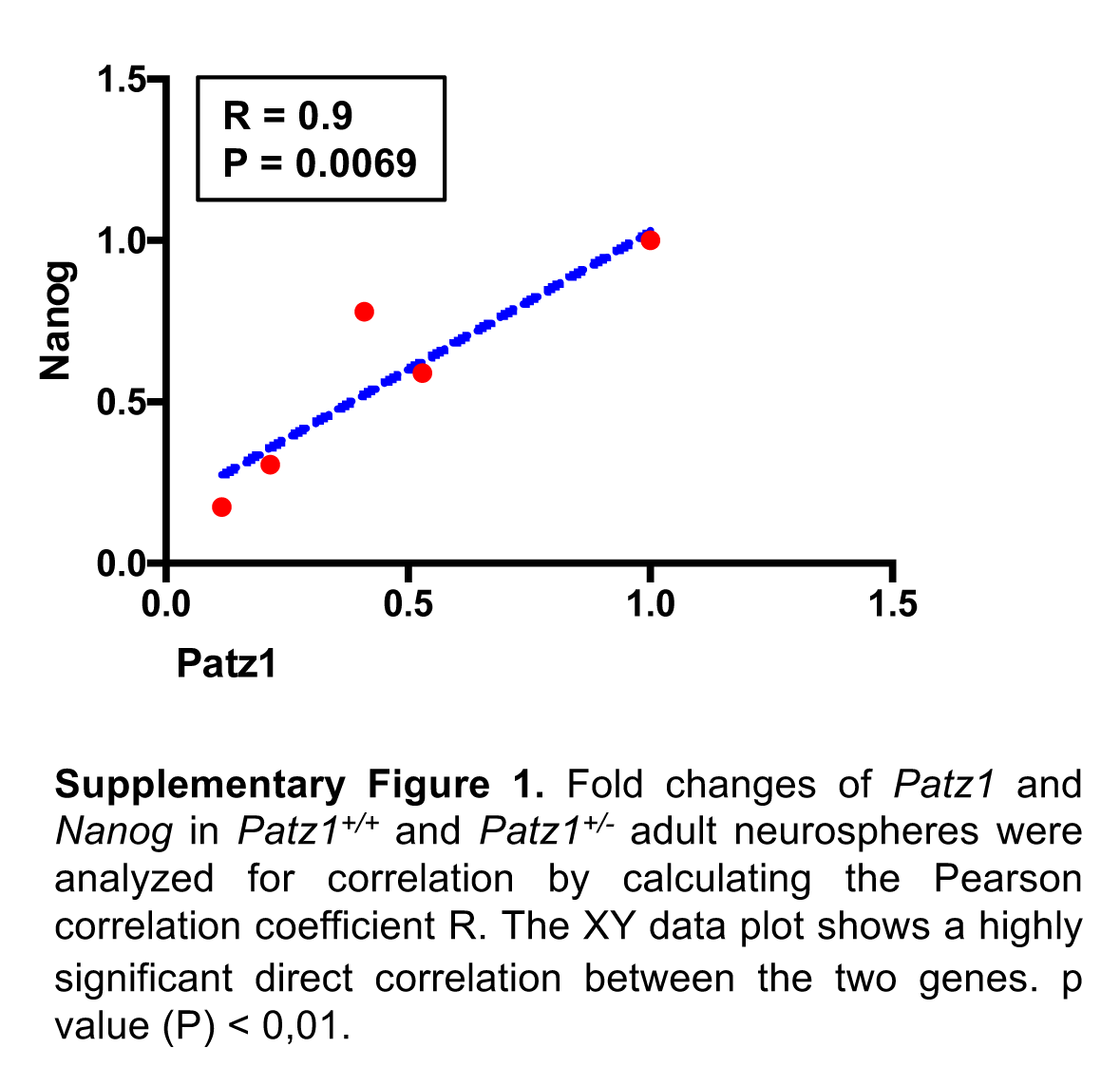

Supplement: Supplementary file 1 [file Image_1.TIF]
